# Amygdala and nucleus accumbens activation are associated with treatment choice in knee osteoarthritis: an fMRI study

**DOI:** 10.1186/s42836-026-00382-x

**Published:** 2026-05-04

**Authors:** Fabiola Ojeda, Gerard Martínez-Vilavella, Laura Blanco-Hinojo, Joan Deus, Laura Tío, Jordi Monfort

**Affiliations:** 1 Cell Research on Inflammation and Cartilage, Programa de Recerca Clínica Translacional, IMIM, Barcelona, 08003 Spain; 2 MRI Research Unit, Department of Radiology, Hospital del Mar, Barcelona, 08003 Spain; 3 Department of Clinical and Health Psychology, Autonomous University of Barcelona, Barcelona, 08193 Spain; 4 Rheumatology Department, Hospital del Mar, Barcelona, 08003 Spain

**Keywords:** Osteoarthritis, Total knee replacement, Central sensitization, Pain catastrophizing, FMRI, Amygdala, Nucleus accumbens

## Abstract

**Background:**

Pain in knee osteoarthritis (KOA) often shows a limited correlation with radiographic severity, complicating clinical assessment and highlighting the relevance of central pain mechanisms. Functional magnetic resonance imaging (fMRI) enables the investigation of brain regions such as the amygdala and nucleus accumbens, which are increasingly recognized as key components of the affective–motivational dimension of chronic pain and may show differential activation across clinical treatment contexts. This study is part of the HOLOA Project (Clinical and virtual examination of patients for holistic and objective description of the osteoarthritis progression mechanisms).

**Methods:**

We conducted a cross-sectional observational study nested within the HOLOA cohort. Thirty-one patients with KOA (20 managed conservatively [CM] and 11 observed in the surgical treatment context) with Kellgren Lawrence (KL) grades 2–3 were included. Participants underwent two fMRI paradigms involving pressure stimulation (Knee Interline and Tibial Surface tests). Clinical assessment included the Western Ontario and McMaster Universities Osteoarthritis Index (WOMAC), Pain Catastrophizing Scale (PCS), Hospital Anxiety and Depression Scale (HADS), and Numeric Rating Scale (NRS). Group comparisons and correlation analyses were performed to examine associations between clinical measures and brain activation patterns.

**Results:**

Groups were broadly comparable with no statistically significant differences in demographic or radiographic severity measures. These patients showed higher WOMAC and PCS scores, indicating greater functional impairment and pain catastrophizing. Across the whole cohort, painful stimulation elicited robust activation of classical pain-processing regions, while no significant amygdala or nucleus accumbens activation was observed at the group level. However, nucleus accumbens activity was positively associated with PCS scores. In between-group analyses, patients observed in the surgical treatment context exhibited significant bilateral amygdala activation during Tibial Surface stimulation, which was absent in the conservatively managed group, and reported higher post-test NRS scores.

**Conclusion:**

Limbic system activation and pain catastrophizing were associated with the surgical treatment context in patients with knee osteoarthritis within a similar range of radiographic severity. The observed involvement of the amygdala and nucleus accumbens underscores the relevance of affective-motivational and cognitive processes in chronic KOA pain. These findings support the value of integrating clinical, psychological, and neurobiological perspectives when interpreting symptom burden and treatment context in knee osteoarthritis.

**Supplementary Information:**

The online version contains supplementary material available at 10.1186/s42836-026-00382-x.

## Background

Osteoarthritis (OA) is the most common chronic joint disorder, characterized by progressive structural damage, pain, swelling, and stiffness [1]. The knee OA (KOA) is the most commonly affected form [2], followed by the hip and hands, and represents a major social burden due to its high prevalence, associated disability, and negative impact on quality of life [3]. Clinical assessment of KOA patients typically involves combining patient-reported questionnaires evaluating pain and function with radiographic imaging [4, 5]. Patients undergoing total knee replacement (TKR) frequently report higher pain intensity and greater psychological distress, such as catastrophizing, compared to those receiving conservative management (CM) [5]. Notably, the decision to undergo surgery is not always associated with more advanced radiographic damage, since radiographic findings often show poor correlation with symptom severity [6–8]. This discrepancy can be explained by the fact that pain in OA arises from a complex interaction between peripheral mechanisms and central adaptations in the nervous system [9]. This clinical complexity likely contributes to the considerable variability observed in surgical decision-making, as TKR is one of the orthopaedic procedures with the highest variation in practice rate [10, 11]. This variation stems primarily from the lack of consensus regarding the functional impairment or the burden of the disease that should warrant surgery. Other factors, such as differences in resource availability across regions, physician beliefs/perspectives about the clinical indications, and patient demand/preferences, also play an important role [11, 12].

To address these challenges, a deeper understanding of the neurobiological mechanisms of KOA pain is required. Functional magnetic resonance imaging (fMRI) provides an objective measure of pain-related brain activity, with evidence showing that central sensitization contributes to pain chronification in many KOA patients [4, 13]. Key limbic structures such as the amygdala and nucleus accumbens, which integrate nociceptive and affective signals, are increasingly recognized as contributors to the persistence of chronic pain [14–17]. Altered activity within these regions may underlie the dissociation often observed between joint damage and symptom severity by amplifying both nociception processing and psychological distress [16–18]. Moreover, several pieces of evidence suggest that OA pain preferentially engages prefrontal limbic rather than somatosensory circuits, reflecting a shift toward centralized emotional processing. These activation patterns have been associated with clinical pain severity, catastrophizing, and reduced efficiency of descending inhibitory control [17, 19, 20]. This study aims to characterize brain activation patterns in patients with knee osteoarthritis, with particular emphasis on the amygdala and nucleus accumbens as key regions involved in the affective–motivational dimension of pain. We examine whether activity in these structures is associated with clinical and psychological measures, and describe the neural correlates of the treatment context by comparing patients managed conservatively with those observed in patients undergoing total knee replacement within a similar range of radiographic severity.

## Methods

### Ethics statement

The study was conducted in accordance with the principles expressed in the Declaration of Helsinki, and the protocol was approved by the Ethical Committee of Clinical Research of the Parc de Salut Mar of Barcelona (ref. MP-TAP-2016-01 and 2016/6747/I) and the Ethics and Institutional Review Board of the Autonomous University of Barcelona (ref. CEEAH-6496). All participants provided written informed consent.

### Study population and clinical assessment

This investigation constitutes a cross-sectional observational sub-study embedded within the framework of the HOLOA Project (Clinical and virtual examination of patients for holistic and objective description of the osteoarthritis progression mechanisms) [5], a larger prospective study with a distinct primary objective (namely, to develop an algorithm capable of delineating clinically relevant phenotypes of KOA).

Clinical assessment included knee radiographs and a comprehensive set of validated patient-reported outcome measures to capture pain intensity, pain mechanisms, functional impact, and psychological factors. Pain and functional impairment were assessed using the Western Ontario and McMaster Universities Osteoarthritis Index (WOMAC) [21], a self-administered questionnaire evaluating pain, stiffness, and physical function. Pain-related catastrophic thinking was measured using the Pain Catastrophizing Scale (PCS) [22**]**. To further characterize pain mechanisms, neuropathic-like pain features were evaluated using questionnaires, culturally adapted and validated for the Spanish population [23]**.** In addition, overall pain severity and its interference with daily activities were assessed using the Brief Pain Inventory (BPI), which provides a multidimensional evaluation of pain intensity and functional impact [24]. Study eligibility criteria required participants to have a confirmed clinical and radiological KOA diagnosis according to the American College of Rheumatology standards [25], incorporating both radiological and clinical assessments, and to present knee symptoms persisting for at least three months prior to screening.

For the present sub-study, eligible participants were individuals aged between 60 and 75 years (we excluded individuals aged over 75 years due to the diminished quality of imaging data in this demographic). Participants should have radiographically established knee osteoarthritis classified as Kellgren and Lawrence (KL) grades 2 or 3 within the past 12 months. Patients from the original HOLOA cohort who met these criteria were invited to participate in the study. Those who agreed to participate provided written informed consent.

Complete eligibility criteria are detailed in Supplementary Table S1**.** Patients from the original HOLOA cohort, who met these criteria, were invited to participate in the study. Those who agreed to participate provided written informed consent. Patient recruitment was conducted between 2016 and 2020 at Hospital del Mar, Barcelona, Spain, by an experienced rheumatologist specialized in OA. In Spain, the decision to proceed with TKR is typically a shared decision-making process between the patient and the physician, primarily based on the severity of pain and functional impairment rather than solely on radiographic findings. Furthermore, structured pre-operative joint replacement education classes are not part of standard pre-operative care. Instead, patients typically receive information about the surgical procedure directly from the orthopaedic surgeon during their clinical consultation. This interaction focuses on explaining the nature of the intervention, expected outcomes, and post-operative care, but does not include standardized psychological or coping-skills training. At the time of the fMRI assessment, all patients included in the TKR group had already been informed of their surgical indication and had agreed to proceed with total knee replacement. Functional MRI examinations were performed after this clinical decision had been communicated to the patient, but before surgery was scheduled or performed. Sample size was originally determined based on the requirements of the HOLOA study and not recalculated for this sub-analysis. A post hoc statistical power analysis for detecting differences in amygdala and nucleus accumbens activation between groups is provided in Supplementary Table S2.

### Functional MRI testing stimuli

Participants underwent two fMRI tests to evaluate brain activation patterns in response to pressure stimulation. In the first test, the Knee Articular Interline test, pressure was applied to the medial articular interline of the knee at the most tender point, with the knee positioned at 60 degrees of flexion. An algometer with a 1 cm^2^ pressure surface delivered painful pressure stimulation of 2.5 kg/cm^2^ at 0.5 Hz in 11 blocks of 10 s each, with five 1-s pulses per block [21, 26–28]. The second test, the Tibial Surface test, involved pressure application to the upper third of the anterior Tibial Surface, 5 cm below the knee, using 4 kg/cm^2^ stimuli in an equal block design. This intensity was chosen based on conventional assessments of primary sensitization disorders [29]. Both tests were conducted over 6 min. Participants rated their subjective pain experience using the Numeric Rating Scale (NRS) [21, 30] immediately after each fMRI sequence.

### Functional MRI acquisition and processing

A Philips Achieva 3.0 Tesla magnet (Philips Healthcare, Best, The Netherlands), equipped with an eight-channel phased-array head coil and single-shot echoplanar imaging (EPI) software, was used for all the fMRI assessments. Functional sequences consisted of gradient recalled acquisition in the steady state (time of repetition [TR], 2,000 ms; time of echo [TE], 35 ms; pulse angle, 70º) within a field of view of 24 cm, with a 64 × 64-pixel matrix, slice thickness of 4 mm (0 mm inter-slice gap) and acquisition voxel size of 3.75 × 3.75 × 4 mm. A total of 32 interleaved slices, parallel to the anterior–posterior commissure line, were acquired to cover the whole brain. The first four (additional) images in each run were discarded to allow the magnetization to reach equilibrium.

Imaging data were processed using MATLAB version 2016a (The MathWorks Inc., Natick, MA, USA) and Statistical Parametric Mapping software (SPM12; The Wellcome Department of Imaging Neuroscience, London, UK). Pre-processing involved motion correction, spatial normalization, and smoothing by means of a Gaussian filter (full-width half-maximum, 8 mm). Data were normalized to the standard SPM-EPI template and resliced to 3 mm isotropic resolution in Montreal Neurological Institute (MNI) space. The functional time series consisted of 180 consecutive image sets obtained over 6 min. All image sequences were visually inspected for potential acquisition and normalization artefacts.

### Functional MRI analysis

Brain activity was analyzed using Statistical Parametric Mapping (SPM12, Welcome Department of Imaging Neuroscience, London, UK) implemented in MATLAB. The goal of the analysis was to identify brain regions that became more active during painful pressure stimulation compared with rest.

To model the brain response, we used a standard approach in fMRI known as the general linear model, which estimates how strongly each brain voxel responds to the stimulus over time. The time course of the stimulation blocks was represented mathematically (a “regressor”) and adjusted for the expected delay in the blood-oxygen-level-dependent (BOLD) signal, which peaks a few seconds after the stimulus [21, 27, 28].

In simple terms, this model allowed us to map which regions of the brain increased their activity during pain and to compare these maps between groups (conservative management vs. TKR). Additional variables such as head motion were included as covariates to reduce noise, and motion-related artefacts were addressed using a within-subject censoring technique (“scrubbing”) [31, 32]. Statistical significance was determined at a voxel-level threshold of *p* < 0.005, corrected for multiple comparisons at *p* < 0.05 (family-wise error). Based on our hypotheses, small-volume corrections were applied to the amygdala and nucleus accumbens to test for localized effects in these regions of interest.

### Statistical analyses

Participant characteristics were compared to assess potential baseline differences between the CM and TKR groups. For categorical variables, Fisher’s exact test was used to compare proportions between groups due to small sample sizes. Continuous variables were analyzed using Student’s *t*-test for normally distributed data or Mann–Whitney U test for non-normally distributed data. Statistical significance was set at *p* < 0.05 for these comparisons.

Brain activation maps for both the Knee Articular Interline and Tibial Surface tests were generated using a one-sample *t*-test. To minimize the impact of motion artifacts, a motion summary measure (mean interframe motion) was included [32] as a nuisance variable for each participant. Group-level comparisons between the CM group and TKR group were performed to identify regions showing differential activation. Additionally, to account for potential confounding by radiographic severity, sensitivity analyses were performed, including Kellgren–Lawrence grade (2 vs 3) as a covariate in the between-group fMRI comparisons. Further analyses examined the whole cohort, sex differences, age-related and body mass index (BMI) correlations, and associations between clinical variables (PCS, WOMAC, HAD, and NRS scores) and brain activation patterns, independent of treatment group. Results were considered significant at a voxel-level threshold of *p* < 0.005 with whole-brain family-wise error correction applied at *p* < 0.05, calculated by means of statistical parametric mapping (SPM). Given their small size and central role in the a priori hypotheses, activation in the amygdala and nucleus accumbens was assessed using small volume correction, applying 5-mm radius spherical masks based on MNI coordinates derived from previous studies [33, 34]. All significant results reported in these regions survived the small volume correction (*p* < 0.05-corrected).

## Results

### Population characteristics and clinical pain assessment

A total of 31 patients were included: 20 patients in the CM group and 11 patients in the TKR group. No statistically significant differences were observed in age, sex distribution, BMI, or Kellgren–Lawrence (KL) grade between groups (Table 1). Although the TKR group showed numerically higher age, BMI, and a greater proportion of KL grade 3, these differences did not reach statistical significance (KL grade distribution, *p* = 0.631).

**Table 1:** Clinical characteristics of the study samples

Characteristic	CM group	TKR group	*p*-value
Sample size (N)	20	11	
Age [years]	64.0 (2.7)	67.1 (4.7)	0.064
Male, N (%)	9 (45)	4 (36)	0.718
BMI [kg/m^2^]	30.6 (4.4)	33.6 (8.1)	0.275
Kellgren-Lawrence degree			
2, N (%)	4 (20.0)	1 (9.1)	
3, N (%)	16 (80.0)	10 (90.9)	0.631
WOMAC score	30.8 (18.4)	50.1 (18.7)	0.012
HADs			
Anxiety	6.9 (4.2)	6.4 (5.0)	0.782
Depression	6.4 (3.4)	6.5 (5.5)	0.957
PCS score	12.0 (9.2)	21.1 (11.3)	0.035
NRS KIt	4.1 (2.6)	4.6 (3.0)	0.647
NRS TSt	5.3 (2.5)	7.3 (2.0)	0.023

Results are expressed as mean (standard deviation) unless otherwise specified. *p*-values were calculated using Welch’s *t*-test for continuous variables and Fisher’s exact test for categorical variables

CM, conservative management; TKR: Total knee replacement; BMI: body mass index; WOMAC: Western Ontario and McMaster Universities Osteoarthritis Index; HAD: Hospital Anxiety and Depression Scale; PCS: Pain Catastrophizing Scale; NRS Kit: Numeric Rating Scale Knee Interline test; NRS TSt: Numeric Rating Scale Tibia Surface test

Pain-related scales showed some differences between groups. The WOMAC scores were significantly higher in the TKR group (*p* = 0.012), reflecting more severe limitations in pain, stiffness, and physical function. Similarly, PCS scores were significantly higher in the TKR group (*p* = 0.035), indicating greater pain-related catastrophic thinking. HAD scores were comparable between groups for both the anxiety and depression domains (Table 1).

### Functional MRI results

**Fig. 1:**
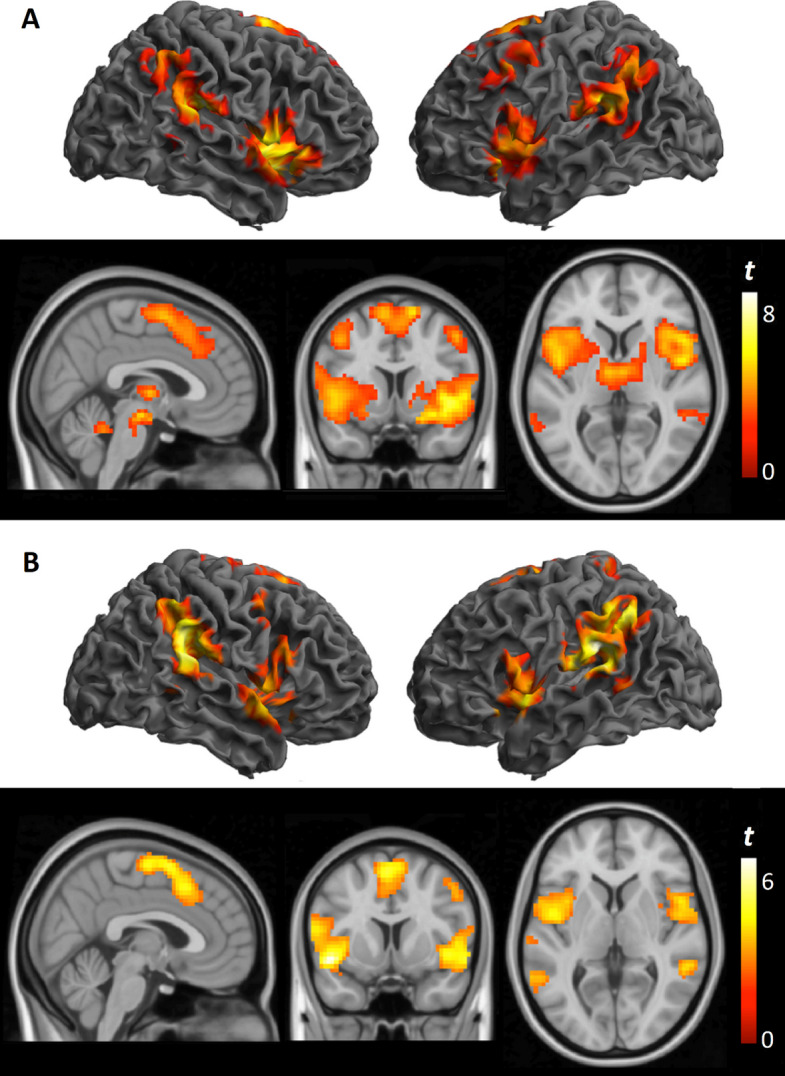
Whole-brain activation maps during pressure stimulation in all patients (*n* = 31). **A** activation map during interline stimulation; (**B**) activation map during tibial surface stimulation. Activations are displayed on a standard MNI brain and projected

To characterize the neural response to pain, we first evaluated the change in brain activation from resting baseline to painful stimulation for the Knee Interline and Tibial Surface tests, across the whole cohort, independent of treatment group. Both stimulation protocols produced robust activation across several brain regions involved in pain processing, such as the somatosensory cortex, supplementary motor area, anterior insula, and anterior cingulate cortex. However, no significant activation was observed in the bilateral amygdala or nucleus accumbens in the total cohort (Fig. 1 and Table 2). Brain activation patterns were similar between Knee Interline and Tibial Surface tests, suggesting an absence of widespread sensitization in the overall cohort.

**Table 2:** Activation maps during pressure stimulation in all patients (*n* = 31)

	**Right hemisphere**		**Left hemisphere**	
	***x, y, z***	***t-value***	***x, y, z***	***t-value***
**Knee interline**				
Lateral temporal cortex	51, − 31, 22	9.4	− 48, − 40, 22	7.7
Insula	42, 14, − 2	8.0	− 45, 14, − 8	5.9
Cerebellum	36, − 64, − 32	7.6	− 33, − 64, − 32	5.5
Prefrontal cortex	48, 8, 46	5.0	− 42, 11, 43	5.1
Supplementary motor area	12, 5, 67	6.2	− 9, 5, 64	5.4
Anterior cingulate cortex	9, 20, 43	5.0		
**Tibial Surface**				
Lateral temporal cortex	51, − 34, 22	6.7	− 57, − 25, 16	6.8
Insula	39, 17, − 11	5.0	− 48, 11, − 11	6.7
Cerebellum	33, − 61, − 32	4.9	− 33, − 61, − 32	6.0
Somatosensory-motor cortex	3, − 10, 64	4.7	− 3, 8, 58	5.0
Anterior cingulate cortex			− 3, − 14, 46	5.0

*x, y, z* are coordinates given in Montreal Neurological Institute (MNI) space

Statistics correspond to a voxel-level threshold of *p* < 0.005, with family-wise error correction at *p* < 0.05. Amygdala and nucleus accumbens results survived small-volume correction

Analysis of sex-dependent activation patterns revealed quantitative differences in neural responses between female and male participants during both stimulation protocols (Supplementary Fig. S1 and Supplementary Table S3). Female participants exhibited more extensive and intense activation across multiple brain regions, but not in the amygdala and nucleus accumbens. However, women reported higher pain intensity in the Knee Interline (2.49, 95% CI, 0.65–4.32, *p*-value: 0.01), but not in the Tibial Surface test (data not shown).

The influence of age on neural activation patterns differed between the two stimulation sites (Supplementary Fig. S2 and Supplementary Table S3). For the Knee Interline test, a significant positive correlation was observed between age and activation intensity in the posterior cingulate cortex, a core element of the default mode network. This pattern was not observed during the Tibial Surface test, where neural responses were relatively consistent across ages.

Analysis of potential correlation between BMI, WOMAC, and HAD with brain activation patterns revealed no significant correlations during either stimulation protocol (data not shown). Activation patterns from baseline to painful stimulation remained consistent across the spectrum of the three variables represented in our study population.

**Fig. 2:**
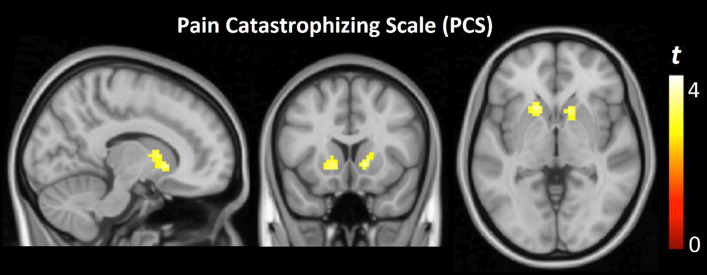
Correlation between Pain Catastrophising Scale (PCS) scores and bilateral nucleus accumbens activation during interline pressure stimulation. The color bar represents *t*-values

There was a significant positive correlation between PCS scores and bilateral activation of the nucleus accumbens during the Knee Interline test (right accumbens: *x* = 12, *y* = 14, *z* = − 5; *t* = 2.9, *p* = 0.004; left accumbens: *x* = − 12, *y* = 17, *z* = − 8; *t* = 3.5, *p* = 0.001) (Fig. 2). Patients reporting higher catastrophizing scores demonstrated increased activation in this region. This correlation was not observed during the Tibial Surface test.

**Fig. 3:**
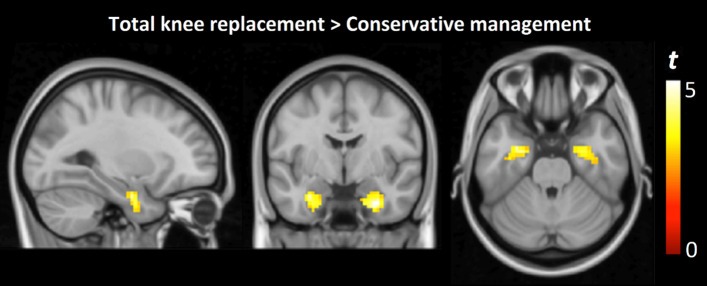
Between-group differences in amygdala activation during tibial pressure stimulation. The color bar represents *t*-values

Comparison of neural activation patterns between CM and TKR patients revealed differences in limbic system activity (Fig. 3). During the Tibial Surface test, TKR patients demonstrated significant bilateral amygdala activation (right amygdala: *x* = 30, *y* = − 4, *z* = − 29; *t* = 4.4, *p* < 0.001; left amygdala: *x* = − 27, *y* = − 7, *z* = − 26; *t* = 4.3, *p* < 0.001), whereas this activation was absent in the CM group. No significant differences between groups were observed during the Knee Interline test. The inclusion of Kellgren–Lawrence grade as a covariate did not materially change the pattern of between-group differences in amygdala activation during the Tibial Surface test. Similarly, patients from the TKR group present significantly higher NRS (2.02, 95% CI, 0.25–3.79, *p*-value = 0.027) only after the Tibial Surface test.

## Discussion

Pain in KOA often shows a limited correlation with radiographic severity, complicating treatment decision-making and highlighting the need to better understand central pain mechanisms. In this context, our study adds novel evidence on the association between limbic system activity and clinical and psychological measures within the treatment context of knee osteoarthritis. Using fMRI, we observed that amygdala and nucleus accumbens activity was associated with clinical and psychological measures and differed between patients managed conservatively and those observed in patients undergoing total knee replacement within a similar range of radiographic severity. Additionally, sex- and age-related differences were identified, with women showing more extensive cortical activation and older participants displaying specific changes in default mode network engagement.

Despite similar demographic profiles, patients in the TKR group exhibited greater functional impairment and catastrophizing, as evidenced by higher WOMAC and PCS scores, compared to those managed conservatively. Neuroimaging revealed no significant activation of amygdala and nucleus accumbens during either the Knee Interline or the Tibial Surface tests in the overall cohort, but patients in the TKR group demonstrated significant bilateral amygdala activation during tibial stimulation, which was absent in the CM group, as observed in the NRS measured after the test. Notably, across the entire cohort, we observed a positive correlation between PCS scores and nucleus accumbens activity, reinforcing the role of affective-motivational processes in the modulation of pain catastrophizing. Emerging neuroimaging evidence highlights the amygdala and nucleus accumbens as pivotal nodes in the transition from acute nociception to chronic pain states, with distinct functional contributions: the amygdala is particularly involved in anticipatory anxiety and fear conditioning, integrating sensory and emotional inputs, while the nucleus accumbens regulates motivational relevance and decision-making under emotionally charged condition through its connectivity with prefrontal cortical regions [35]. These neurobehavioral distinctions may help contextualize why some patients are observed in the surgical treatment context despite comparable structural damage, as limbic system dysregulation could bias risk-reward evaluations and amplify the perceived need for surgery [36].

Importantly, all patients in the TKR group were aware of their surgical indication at the time of the fMRI assessment. Accordingly, the observed limbic activation should be interpreted as a neural correlate of the clinical and emotional context in which the treatment decision was made, rather than as a causal driver of surgical choice. The cross-sectional design and the timing of the fMRI assessment preclude causal inference, and the findings may reflect state-dependent emotional processes such as anticipatory anxiety, expectancy, or perceived threat related to impending surgery.

This heightened amygdala activity, together with elevated psychological distress scores, was observed in patients who ultimately underwent surgical intervention, suggesting that neural and psychological changes are already present in patients selected for surgical intervention. Importantly, these patients are not necessarily those with greater objective joint dysfunction or intractable pain, but rather those patients who may be more likely to seek or accept surgical intervention, potentially driven by altered sensory processing and emotional dysregulation. In fact, recent findings have shown an association between higher PCS scores and belonging to the TKR group, supporting the notion that catastrophizing may influence surgical decision-making independently of structural severity [5]. They may be at risk of persistent symptoms after surgery, as suggested by prior literature, since their symptoms are more centrally mediated and may persist after surgery. Consistent with this interpretation, higher PCS have been associated with poorer outcomes and lower satisfaction after TKR, despite comparable structural damage. Darnall et al. [37] described that PCS predicts the worst post-surgical pain outcomes, with reported effect sizes reaching 2.37. Similarly, multifactorial analyses of primary TKR revealed that dissatisfied patients had significantly higher pre-operative PCS (*p* = 0.03) and lower KL grade compared to satisfied patients [37, 38]. The observed association between amygdala activation and surgical status suggests that dysfunctional emotional pain regulation characterizes patients observed in the surgical treatment context and may be relevant for understanding vulnerability to poor outcomes rather than surgical need per se. These findings align with previous research from Pujol et al. showing altered insula function during rest [39], underscoring the need to integrate both emotional and sensory dimensions of pain into OA treatment planning.

Sex- and age-dependent differences in brain activation were also seen, with females showing more extensive activation associated with higher pain scores, only in the Knee Interline test, and older participants exhibiting increased activation during the Knee Interline test, despite reporting similar pain intensity levels. These results are consistent with previous neuroimaging studies showing that women exhibit stronger and more widespread activation in pain-processing regions compared to men [40]. Such differences have been linked to both biological factors, such as hormonal modulation of nociceptive pathways, and psychosocial influences on pain perception. Stronger cortical recruitment in females may reflect greater affective–cognitive engagement with pain stimuli. Our findings extend this evidence to KOA, highlighting sex-related differences in chronic musculoskeletal pain processing. These findings are consistent with previous literature and were accompanied by higher WOMAC scores among female participants in our cohort [5], suggesting greater perceived disability and functional impact. The absence of sex differences in amygdala or nucleus accumbens activation indicates that these effects may reflect broader cortical involvement rather than limbic-specific effects. Together, these results support the notion that sex-related differences in KOA pain may arise primarily from central mechanisms involved in pain modulation and emotional appraisal, rather than from peripheral nociceptive input alone. Clinically, this highlights the need for sex-tailored approaches targeting cognitive and emotional dimensions of pain rather than solely focusing on joint-level interventions.

The divergent age effects between stimulation sites underscore the context-specific nature of pain-related brain responses. The positive correlation between age and posterior cingulate cortex activation during the Knee Interline test suggests that advancing age may enhance recruitment of default mode network regions in response to pain. This association does not appear to reflect greater radiographic severity, but may instead relate to longer disease duration and the cumulative impact of chronic pain on central processing, as previously suggested in chronic musculoskeletal conditions [41]. The absence of age-related effects in the Tibial Surface test highlights the stimulus-specificity of these adaptations. This study has several limitations that should be acknowledged. Given the number of exploratory analyses performed, results should be interpreted as hypothesis-generating. First, its cross-sectional design precludes any causal inference regarding the relationship between limbic activation and treatment choice. Functional MRI assessments were performed after patients in the TKR group had already been informed of their surgical indication; therefore, the observed brain activation patterns may reflect state-dependent emotional processes, such as anticipatory anxiety, expectancy, or situational stress related to impending surgery, rather than pre-existing neural traits. Accordingly, limbic activation should be interpreted as a neural correlate of the clinical and emotional context in which the treatment decision was made, rather than as a causal driver of surgical choice.

Second, the small sample size, particularly in the male TKR subgroup, limited statistical power and precluded robust subgroup or stratified analyses, and effect sizes may therefore be inflated. Third, treatment decisions for TKR vary substantially across healthcare systems and countries, with different thresholds and criteria for surgical intervention. These variations in clinical practice patterns may restrict the generalizability of our findings. For example, while Spanish guidelines emphasize functional impairment as measured by WOMAC scores, other healthcare systems may prioritize different clinical parameters or apply distinct decision-making algorithms. Furthermore, although radiographic severity was largely comparable between groups, residual confounding related to structural disease burden cannot be entirely excluded, despite sensitivity analyses adjusting for KL grade.

Despite these limitations, the study also has notable strengths. It integrates advanced fMRI paradigms with comprehensive clinical and psychological assessment, allowing the characterization of neural activation patterns associated with pain sensitization within a real-world treatment context. The comparison between conservatively managed patients and those observed in the surgical treatment context provides valuable insight into how limbic system activity and psychological factors relate to symptom burden beyond structural disease severity.

The brain activation patterns identified in this study open potential avenues for integrated therapeutic strategies that address both joint pathology and central pain modulation. In particular, heightened activity in limbic structures such as the amygdala and nucleus accumbens was observed in patients within the surgical treatment context and was associated with elevated pain catastrophizing. As catastrophizing represents a modifiable psychological factor, these findings support the rationale for targeted pre-operative interventions aimed at addressing central sensitization and maladaptive affective–motivational pain processing rather than structural disease severity alone. Evidence supports the efficacy of brief psychological interventions, including cognitive behavioral therapy, mindfulness-based approaches, and pain neuroscience education, in reducing catastrophizing, improving pain-related outcomes, and enhancing patient satisfaction following total knee replacement. In addition, pharmacological agents with central modulatory properties, such as duloxetine or gabapentinoids, which have been shown to influence limbic activity and descending pain inhibition pathways, may represent valuable adjunctive treatments in patients exhibiting features of central sensitization. Together, these strategies may help optimize symptom management and patient stratification within a multimodal, personalized care framework, while underscoring the importance of addressing central pain mechanisms alongside peripheral joint pathology [42, 43].

Furthermore, non-invasive neuromodulator interventions (such as transcranial magnetic stimulation or transcutaneous vagus nerve stimulation) may offer targeted modulation of hyperactive limbic circuits in select patients.

Age-related alterations in brain networks, such as the default mode network, further support the utility of mindfulness approaches in older adults by enhancing interoceptive awareness and emotional regulation. Despite these promising avenues, psychological screening and central pain–focused interventions remain largely absent from standard orthopaedic practice. Incorporating central pain assessments and PCS evaluation into routine pre-operative workups could help stratify surgical patients and guide multimodal, individualized treatment strategies better aligned with the neurobiological complexity of OA pain. Accordingly, limbic hyperactivity should be interpreted as a correlate of the clinical decision-making context rather than as a definitive causal driver of surgical choice. The cross-sectional design and the fact that TKR patients were already aware of their surgical indication at the time of scanning limit causal inferences regarding whether limbic activation precedes or results from treatment decision-making.

Future studies should investigate whether amygdala hyperactivity during painful stimulation predicts clinical outcomes and treatment response. Exploring the efficacy of brain-targeted interventions in KOA patients with central sensitization could pave the way for a more personalized and effective approach to pain management.

## Conclusions

In this cross-sectional fMRI study of patients with knee osteoarthritis, we identified differences in limbic system activation patterns between patients managed conservatively and those observed in the surgical treatment context, despite a similar range of radiographic severity. Patients undergoing total knee replacement showed greater amygdala reactivity to painful stimulation, together with higher WOMAC and pain catastrophizing scores, reflecting increased affective–motivational engagement with pain. Across the overall cohort, nucleus accumbens activation was positively associated with catastrophizing, supporting the relevance of emotional and cognitive processes in chronic pain experience.

These findings suggest that treatment context in knee osteoarthritis is associated not only with structural and functional measures, but also with central pain-related neural activity. Limbic system activation, particularly within the amygdala, appears to characterize patients experiencing higher psychological and affective burden in the setting of advanced symptom severity, rather than reflecting radiographic progression alone.

Importantly, catastrophizing emerged as a modifiable psychological factor associated with limbic activation. This observation supports the potential value of incorporating psychological assessment and central pain evaluation into the comprehensive clinical assessment of patients with knee osteoarthritis. Such an integrative perspective may help better contextualize symptom burden and guide future research aimed at optimizing multimodal, individualized management strategies.

Overall, our results highlight the importance of considering clinical, psychological, and neurobiological dimensions together when interpreting pain severity and treatment context in knee osteoarthritis. Longitudinal and prospective studies will be required to determine the temporal relationship between limbic activation, psychological factors, and treatment outcomes.

## Supplementary Information

Supplementary Material 1.

## Data Availability

The datasets generated and analysed during the current study are available from the corresponding author upon reasonable request. Due to ethical and confidentiality restrictions, the neuroimaging data and clinical datasets cannot be made publicly available.

## Abbreviations

ACR: American College of Rheumatology
ACC: Anterior cingulate cortex
AEI: Agencia Estatal de Investigación (Spanish State Research Agency)
BOLD: Blood-oxygen-level dependent
BMI: Body mass index
CBT: Cognitive behavioral therapy
CM: Conservative management
DMN: Default mode network
fMRI: Functional magnetic resonance imaging
HAD: Hospital Anxiety and Depression Scale
IQR: Interquartile range
KL: Kellgren–Lawrence
KOA: Knee osteoarthritis
MNI: Montreal Neurological Institute
MRI: Magnetic resonance imaging
NAcc: Nucleus accumbens
NRS: Numeric Rating Scale
OA: Osteoarthritis
PCS: Pain Catastrophizing Scale
ROI: Region of interest
SD: Standard deviation
SPM: Statistical Parametric Mapping
SYSADOA: Symptomatic slow-acting drugs for osteoarthritis
TKR: Total knee replacement
TR: Time of repetition
TE: Time of echo
WOMAC: Western Ontario and McMaster Universities Osteoarthritis Index
