# Supplementary material for: Amygdala and nucleus accumbens activation are associated with treatment choice in knee osteoarthritis: an fMRI study
Source: Arthroplasty. 2026 May 4;8:32. doi: 10.1186/s42836-026-00382-x (PMC13137494; doi:10.1186/s42836-026-00382-x)
Supplement: Supplementary file 1 — Supplementary Material 1. [file 42836_2026_382_MOESM1_ESM.docx]

**Supplementary information**

**Table S1.** List of patient eligibility criteria

| **Inclusion criteria** | **Exclusion criteria** |
| --- | --- |
| - Fulfillment of the American College of Rheumatology (ACR) classification criteria for clinical Knee OA. - Aged between 60 and 70 years. - Knee radiography (performed in the previous 12 months) graded 2 or 3 according to Kellgren and Lawrence (KL) scale. - Presence of symptomatology (pain, dysfunction, and/or effusion) in the last 3 months. - Ability to provide written informed consent. | - OA presence exclusively in either the lateral femorotibial or the patellofemoral compartments. - Presence of secondary OA:   - Partial or total meniscectomy.   - Inflammatory or connective tissue diseases.   - Overuse of the joint from work or sporting activities.   - Pathological varus or valgus deformity.   - Presence of microcrystals in the articular space. - Fibromyalgia. - Underlying uncompensated health condition. - Need for assistance or support to walk (crutch, walker). - MRI contraindications. - Use of the following OA treatments in the indicated period prior to the clinical visits:   - NSAIDs: 1 week.   - Morphics: 1 week.   - Corticosteroids: 1 month.   - SYSADOA: 2 months.   - Intraarticular hyaluronic acid: 3 months. - Abuse of substances in the 6 months prior to the study |

**Table S2.** Statistical power analysis for detecting amygdala and nucleus accumbens activation differences, with an alpha level of 0.05 in a two-tailed test and sample sizes of 20 and 11 participants, respectively

|  | Statistical power | SD | Mean CM group | Mean TKR group |
| --- | --- | --- | --- | --- |
| Amygdala_Left | 87% | 0.509 | −0.328 | 0.260 |
| Amygdala_Right | 90% | 0.385 | −0.396 | 0.069 |
| Nucleus accumbens_Left | 8% | 0.332 | −0.001 | 0.061 |
| Nucleus accumbens_Right | 3% | 0.341 | 0.054 | 0.052 |

CM, conservative management; TKR: Total knee replacement; SD: standard deviation.

**Table S3.** Sex and age differences in activation patterns during pressure stimulation

|  | **Cluster-level** | | | **Peak-level** | | |
| --- | --- | --- | --- | --- | --- | --- |
|  | **Voxels** | **P_FWE-corr_** | | ***x, y, z*** | ***t*-value** | ***p*-value** |
| **Sex (women > men)** |  | |  |  |  |  |
| **Knee interline** |  |  | |  |  |  |
| Right occipital cortex | 3327 | <0.001 | | 21, −85, 22 | 5.6 | <0.001 |
| Left occipital cortex | sc | sc | | −21, −85, 22 | 4.6 | <0.001 |
| Cerebellum | sc | sc | | 12, −64, −29 | 4.4 | <0.001 |
| **Tibial surface** |  |  | |  |  |  |
| Motor cortex | 450 | 0.010 | | −42, −7, 61 | 5.3 | <0.001 |
| Cerebellum | 493 | 0.007 | | 33, −61, −23 | 4.1 | <0.001 |
| **Age (positive correlation)** |  |  | |  |  |  |
| **Knee interline** |  |  | |  |  |  |
| Posterior cingulate cortex | 464 | 0.008 | | 0, −49, 19 | 4.9 | <0.001 |

*x, y, z*: coordinates given in Montreal Neurological Institute (MNI) space. Statistics correspond to a corrected threshold P_FWE_ (*PFWE-corr)* < 0.05 estimated using SPM. sc: same cluster.

**Supplementary**


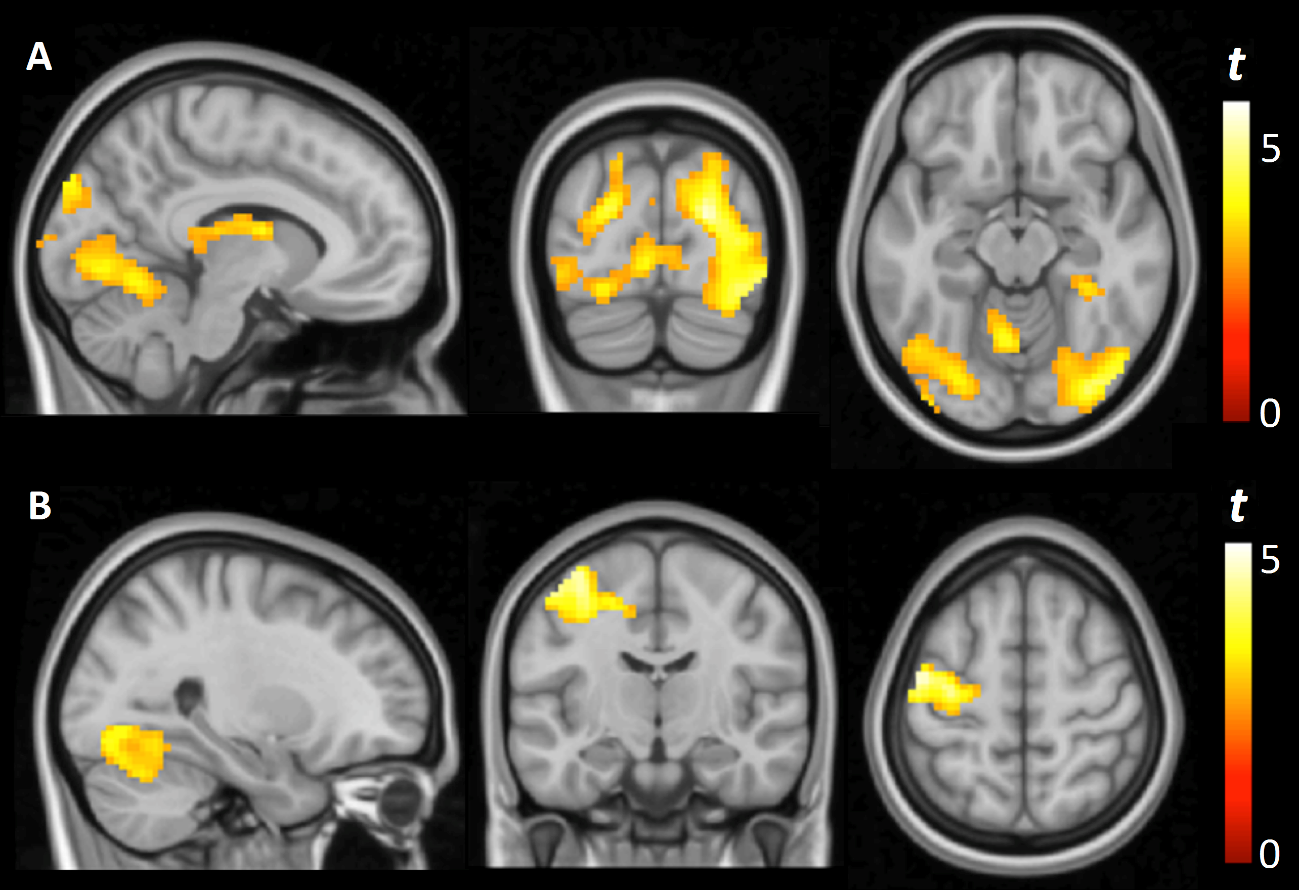


**Fig. S1.** Sex differences in brain activation during pressure stimulation. The figure illustrates regions where women participants showed increased activation relative to men. (**A**) knee interline; (**B**) tibial surface. Color bars represent *t*-values.


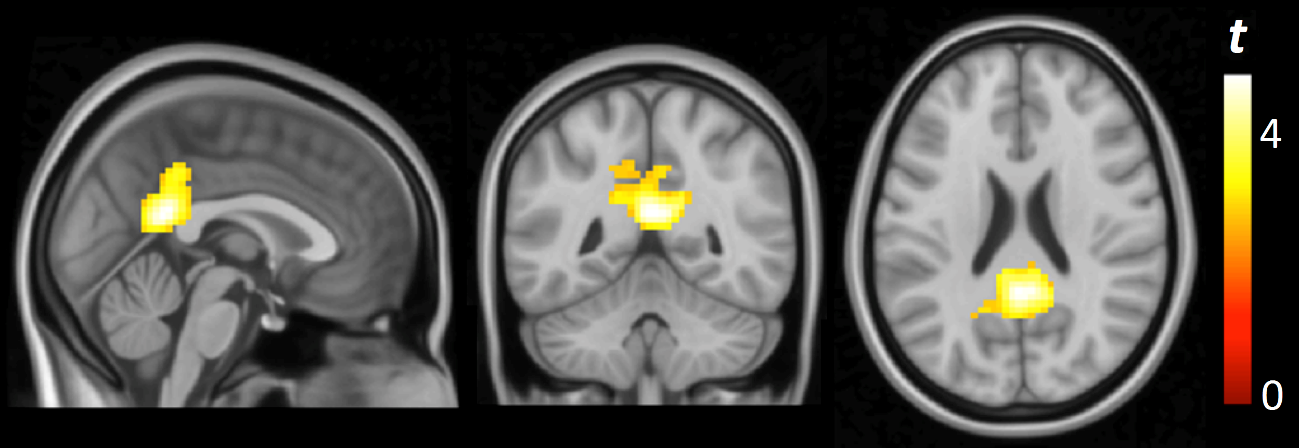


**Fig. S2.** Age-related effects on brain activation during knee interline pressure stimulation. The figure illustrates the brain region where activation increases with age across participants. The color bar represents *t*-values.
